# Isopropyl‐phloroglucinol‐DHA protects outer retinal cells against lethal dose of all‐*trans*‐retinal

**DOI:** 10.1111/jcmm.15135

**Published:** 2020-03-25

**Authors:** Aurélie Cubizolle, David Cia, Espérance Moine, Nathalie Jacquemot, Laurent Guillou, Mélissa Rosell, Claire Angebault‐Prouteau, Guy Lenaers, Isabelle Meunier, Joseph Vercauteren, Thierry Durand, Céline Crauste, Philippe Brabet

**Affiliations:** ^1^ INSERM U1051 Institut des Neurosciences de Montpellier Montpellier France; ^2^ Université Montpellier Montpellier France; ^3^ UMR INSERM 1107 Laboratoire de Biophysique Neurosensorielle Facultés de Médecine et de Pharmacie Clermont‐Ferrand France; ^4^ UMR5247‐CNRS‐UM ENSCM Faculté de Pharmacie Institut des Biomolecules Max Mousseron (IBMM) Montpellier France; ^5^ INSERM U1046 UMR CNRS 9214 CHRU de Montpellier Montpellier France; ^6^ INSERM U1083 CNRS UMR 6015 MitoVasc-MitoLab Université d'Angers Angers France; ^7^ National Reference Centre for Inherited Sensory Disorders, CHU Montpellier France

**Keywords:** all‐*trans*‐retinal, bisretinoid A2E, carbonyl stress, isopropyl‐phloroglucinol‐DHA conjugate, outer retina cells, oxidative stress

## Abstract

All‐*trans*‐retinal (a*t*RAL) is a highly reactive carbonyl specie, known for its reactivity on cellular phosphatidylethanolamine in photoreceptor. It is generated by photoisomerization of 11‐*cis*‐retinal chromophore linked to opsin by the Schiff's base reaction. In ABCA4‐associated autosomal recessive Stargardt macular dystrophy, a*t*RAL results in carbonyl and oxidative stress, which leads to bisretinoid A2E, accumulation in the retinal pigment epithelium (RPE). This A2E‐accumulation presents as lipofuscin fluorescent pigment, and its photooxidation causes subsequent damage. Here we describe protection against a lethal dose of a*t*RAL in both photoreceptors and RPE in primary cultures by a lipidic polyphenol derivative, an isopropyl‐phloroglucinol linked to DHA, referred to as IP‐DHA. Next, we addressed the cellular and molecular defence mechanisms in commonly used human ARPE‐19 cells. We determined that both polyunsaturated fatty acid and isopropyl substituents bond to phloroglucinol are essential to confer the highest protection. IP‐DHA responds rapidly against the toxicity of a*t*RAL and its protective effect persists. This healthy effect of IP‐DHA applies to the mitochondrial respiration. IP‐DHA also rescues RPE cells subjected to the toxic effects of A2E after blue light exposure. Together, our findings suggest that the beneficial role of IP‐DHA in retinal cells involves both anti‐carbonyl and anti‐oxidative capacities.

## 1 INTRODUCTION

In vertebrates, visual perception occurs in the rod and cone photoreceptor outer segments (POS) through visual pigments, G protein‐coupled receptors (rhodopsin and cone opsins) consisting of the apoprotein opsin and a prosthetic group, the vitamin A‐derived chromophore 11‐*cis*‐retinal (11*c*RAL).^1^ The light isomerizes 11*c*RAL to *trans* configuration (a*t*RAL) and subsequently elicits the conformational transition within the opsin proteins, followed by the activation of the phototransduction cascade. After photoisomerization of 11*c*RAL to a*t*RAL in POS, the a*t*RAL Schiff base is hydrolysed, yielding the photochemically inactive opsin protein and the freed a*t*RAL.^2^ Removal of the latter is required to avoid acute toxicity, and delayed clearance of a*t*RAL after light exposure contributes to light‐induced retinal degeneration.^3^ The a*t*RAL is cleared from disc membranes of POS by retinal ATP‐binding cassette (ABCA4) transporter proteins to the cytoplasm where a*t*RAL‐dehydrogenase (RDH) catalyses its reduction to the much less reactive all‐*trans*‐retinol (a*t*ROL). Mutations in the *ABCA4* gene are found in patients with Stargardt macular dystrophy (STGD1), cone‐rod dystrophy and recessive retinitis pigmentosa, and variants in ABCA4 increased susceptibility to age‐related macular degeneration (AMD).^4^

Mechanisms of acute toxicity of a*t*RAL were previously studied by Palczewski and coworkers.^3^, ^5^ They first reported that NADPH oxidase at the plasma membrane can be activated by an increase in a*t*RAL levels via the phospholipase C/ inositol 1,4,5‐triphosphate pathway, resulting in overproduction of reactive oxygen species (ROS). The respiratory chain in mitochondria also participates in ROS production (the more reactive being HO^·^) within the cell in reply to a*t*RAL accumulation.^3^ More recently, a*t*RAL has been shown to induce mitochondrial transmembrane potential loss and endoplasmic reticulum (ER) stress that ultimately trigger programmed cell death by activating apoptotic Bax‐ and caspase‐dependent cascades.^6^, ^7^ Free a*t*RAL is itself a reactive carbonyl compound through its all *trans‐*polyene conjugated aldehyde that is toxic to cells.^8^, ^9^

In *ABCA4*‐associated pathologies, a*t*RAL accumulates due to delayed clearance by the defective ABCR transporter. Nickell et al^10^ reported that rhodopsin is present at a concentration of 4.62 mmol/L in disc membranes of rod outer segments. Therefore, the level of a*t*RAL released after photoactivation of rhodopsin can range from 25 to 100 μmol/L in the disc membranes following photobleaching of only 0.5%‐2%. This level of a*t*RAL is toxic in cultured retinal cells.^7^, ^9^ Excess a*t*RAL is a potent photosensitizer which can mediate light‐induced oxidation.^11^ However, a*t*RAL condenses on the PE by a double mechanism of carbonyl and oxidative stress.^12^, ^13^ This leads to decrease a*t*RAL levels and to the formation of bisretinoid adducts such as A2E and RAL dimer, which are pigments of retinal pigment epithelium (RPE) autofluorescent lipofuscin.^14^ These pigments are sensitive to visible blue light and are photo‐oxidized and fragmented accordingly.^15^ The oxidized metabolites are reactive carbonyl and oxidative species that would have toxic effects in the RPE.^16^

Based on epidemiology studies, natural antioxidants such as polyphenols appear as efficient protectors against oxidative stress. This activity may be related to their capacity to block the formation and accumulation of ROS or to stimulate the enzymatic antioxidant defences of the organism.^17^ Literature also addressed the efficiency of polyphenols to act as anti‐carbonyl stressor agents by trapping reactive toxic carbonyl entities.^18^ We previously reported in vitro cytoprotective effects of the polyphenol phloroglucinol, a natural monomer of phlorotannins abundantly present in Ecklonia cava (edible brown algae), in outer retinal cells by scavenging ROS and trapping a*t*RAL.^9^ Because of its low bioavailability, phloroglucinol was then structurally modified by the addition of polyunsaturated fatty acid (PUFA) and isopropyl substituents.^13^

In the present study, we investigated the protective effect of the medicinal chemical compound, isopropyl‐phloroglucinol‐DHA (IP‐DHA), also called lipophenol, against a*t*RAL‐related carbonyl and oxidative stresses (COS). We first analysed primary cultures of outer retina to demonstrate the dose‐dependent protective effect against lethal dose of a*t*RAL. We then used ARPE‐19 cells as a standard cellular model to study the mechanisms of cell death and protection. We demonstrate that each of the structural part, that is isopropyl and PUFA, is essential for the full action of the lipophenol with selectivity for LA and DHA. We compared the capacities of phloroglucinol and IP‐DHA to reverse the effects of a*t*RAL and finally discuss how to understand the enhanced protective effects of IP‐DHA compared to phloroglucinol.

## 2 MATERIALS AND METHODS

### 2.1 Chemicals

All‐*trans* retinal (a*t*RAL), 3‐(4,5‐dimethylthiazol‐2‐yl)‐2, 5‐diphenyl tetrazolium bromide (MTT) and 2′,7′dichlorofluorescin diacetate (H_2_DCFDA) were purchased from Sigma‐Aldrich. A*t*RAL was dissolved in dimethylformamide (DMF) and freshly diluted in serum‐free culture medium to working concentrations in 0.1% DMF. MTT and H_2_DCFDA were used at 0.5 mg/mL and 2 μmol/L, respectively. A2E was synthesized as previously described.^19^

### 2.2 Synthesis of phloroglucinol lipophenols

To evaluate the influence of different lipid chains and of the isopropyl substituent, several lipophenols were synthesized: five lipophenols with an isopropyl‐phloroglucinol (IP) core linked to various fatty acid, IP‐DHA, IP‐EPA, IP‐ALA, IP‐LA and IP‐C22, and three lipophenols using only the phloroglucinol without alkyl substituent, P‐DHA, P‐EPA and P‐LA. All the lipophenols were synthesized according to the chemical strategy developed by Crauste et al.^13^ Briefly, one hydroxyl group of the phloroglucinol or IP is protected by triisopropylsilyl (TIPS) groups using triflate reagent (TIPS‐OTf) and diisopropylethylamine (DIPEA) as a base to obtain the protected derivative. The coupling reactions between the protected polyphenol and the different fatty acids, docosahexaenoic acid (DHA), eicosapentaenoic acid (EPA), α‐linolenic acid (ALA), linoleic acid (LA) and behenic acid (C22), were initiated using dicyclohexylcarbodiimide and dimethylaminopyridine (DCC/DMAP) as coupling reagents to access the protected lipophenols. Deprotection of the TIPS groups by Et_3_N‐3HF in dry tetrahydrofuran (THF) yielded final lipophenols compounds, IP‐DHA, IP‐EPA, IP‐ALA, IP‐LA, IP‐C22, P‐DHA, P‐EPA and P‐LA**.** A quality control assessment was established by a complete ^1^H and ^13^C NMR spectral analysis for each synthesized compound (chemical structure, general procedure, yield and NMR analysis are reported in Supporting Information).

### 2.3 Cell cultures and a*t*RAL treatment

Primary rat RPE and mouse neural retina (NR) cultures were obtained as previously described.^9^ RPE cells were cultured for 3 days until they reached 80%‐85% confluency, and NR was cultured for 10 days until glial cells were confluent in the bottom layer and neural cells with a neurite outgrowth in the upper layer. Human RPE‐like cells, ARPE‐19, were seeded at 100 000 cells/cm^2^ and grown to confluency before being assayed as instruct by ATCC.

Pre‐ and co‐treatment procedures with lipophenol were carried out. During pre‐treatments, rat RPE primary cultures received a medium containing lipophenol at different concentrations (40‐320 μmol/L) for 24 hours. The medium was then removed and replaced by a serum‐free culture medium containing 25 μmol/L a*t*RAL for 4 hours. During co‐treatments, RPE and NR cells received a serum‐free medium containing 25 μmol/L a*t*RAL and/or IP‐DHA (10‐320 μmol/L) for 4 hours. NR cells were refreshed with serum‐free medium the next day.

### 2.4 A2E treatment

ARPE‐19 cells were plated into 96‐well plates (4 × 10^4^ cells/well) and cultured for 24 hours to confluence prior to lipophenol treatment. The cell cultures were treated with serum‐free DMEM/F12 medium without phenol red containing IP‐DHA at different concentrations (0‐80 μmol/L) for 1 hour. A2E was then added to a final concentration of 20 μmol/L for 6 hours before rinsing with medium. Control cells were incubated with 0.2% DMSO with or without A2E. The cells were exposed to intense blue light (4600 lux) for 30 minutes to induce phototoxicity of A2E and incubated at 37°C. Irradiation was achieved using a LED device with blue emission wavelengths from 430 to 470 nm and a dimmable luminance (Roleadro lighting). Control ambient white light was less than 300 lux. Without blue light exposure, A2E‐loaded cell survival was not affected. The cell viability was determined 16‐20 hours later using a MTT colorimetric assay. Results are expressed in percentage of viable cells normalized to control conditions in the absence of lipophenol and stressor.

### 2.5 Cell viability

Cell viability in primary RPE and ARPE‐19 was determined by MTT assay in 96‐well plates as described.^9^ To distinguish viable cells from apoptotic and necrotic cells, treated ARPE‐19 were cultured in 24‐well plates and stained with Annexin V (A)—FITC and propidium iodide (PI). Cells were analysed by a BD‐LSRII flow cytometer with FACSDiva Software (BD Biosciences) at the Cellular Health Imaging Center of Clermont Auvergne University. Living cells were first sorted according to their size (FSC) and granularity (SSC). Cell states were identified as follow: living cells (A−, PI−), early apoptotic cells (A+, PI−) and late apoptotic/necrotic cells (A+, PI+).

### 2.6 Mitochondrial respirometry

Mitochondrial respiration was measured in ARPE‐19 cultured in six‐well plates after 4‐hour exposure to 25 μmol/L a*t*RAL and/or 40 μmol/L lipophenol. Respiration was measured on 10^6^ cells permeabilized by incubation for 2 minutes with 15 µg digitonin and resuspended in a respiratory buffer (pH 7.4, 10 mmol/L KH_2_PO_4_, 300 mmol/L mannitol, 10 mmol/L KCl and 5 mmol/L MgCl_2_). The respiratory rates were recorded at 37°C in 2‐mL glass chambers using a high‐resolution Oxygraph respirometer (Oroboros) as recently described.^20^ Assays were initiated in the presence of 5 mmol/L malate/pyruvate to measure basal respiration. (state 2), Complex I‐coupled state 3 respiration was measured by adding 0.5 mmol/L NAD^+^/1.5 mmol/L ADP. Then, 10 mmol/L succinate was added to reach maximal coupled respiration, and 10 μmol/L rotenone was injected to obtain the CII‐coupled state 3 respiration. Oligomycin (8 µg/mL) was added to determine the uncoupled state 4 respiration rate. Finally, carbonyl cyanide‐4‐(trifluoromethoxy) phenylhydrazone (1 μmol/L) was added to control the permeabilization of the tissues.

The respiration rate driven by complex IV was measured starting from CII‐coupled state 3 and the addition of antinomycin A (1 μmol/L), which inhibited complex III, and of ascorbate/TMPD redox couple to reduce cytochrome c.

### 2.7 Mitochondrial enzymatic activities

The enzymatic activity of the mitochondrial respiratory chain complexes (RCC) was measured on cell homogenates as described previously.^21^ Briefly, ARPE‐19 was grown in six‐well plates and treated for 4 hours with 25 μmol/L a*t*RAL and/or 40 μmol/L lipophenol. Cells were scraped, rinsed with DPBS and resuspended on ice with cell buffer (250 mmol/L saccharose, 20 mmol/L tris[hydroxymethyl]aminomethane, 2 mmol/L EGTA, and 1 mg/mL bovine serum albumin (BSA), pH 7.2). Cell was disrupted by two freezing‐thawing cycles, centrifuged at 16 000 *g* for one minute and suspended in the cell buffer (50 µL/10^6^ cells). The cellular protein content was determined with the Bicinchoninic assay kit (Pierce) using BSA as standard. The initial kinetics of enzymatic activities were monitored by spectrophotometry (UV‐SAFAS spectrophotometer, SAFAS monaco). Complex I (NADH ubiquinone reductase) activity was measured as described elsewhere^22^ and adapted using 2, 6 dichloroindophenol (DCPIP) to avoid inhibition of complex I activity by decylubiquinol.^23^ Complex II (succinate ubiquinone reductase) activity was measured according to James et al.^24^ Specific enzymatic activities of complexes I and II were expressed in mIU (ie nanomoles of DCPIP/min/mg protein). Complex IV (cytochrome c oxidase) activity was recorded according to a method by Rustin et al,^25^ adapted in a 50 mmol/L KH_2_PO_4_ buffer, using 15 μmol/L reduced cytochrome *c*. Specific enzymatic activity was expressed in mIU (ie nanomoles of cyt *c*/min/mg protein). Citrate synthase activity was assayed using a standard procedure.^26^ Specific enzymatic activity was expressed in mIU (ie nanomoles of 5‐5′‐dithiobis (2‐nitrobenzoic acid), DTNB/min/mg protein).

### 2.8 ROS production

ROS were measured in ARPE‐19 using the H_2_DCFDA probe as described^9^ with minor modifications. Radicals such as peroxyl, alkoxyl, NO_2_^·^, carbonate or HO^·^ are able to oxidize H_2_DCFDA and thus to be quantified by this assay.^14^, ^27^ Briefly, cells seeded on black, optically clear‐bottom 96‐well plates (Perkin‐Elmer) were incubated with 2 μmol/L H_2_DCFDA in phenol red free DMEM/F12 for 45 minutes, washed with PBS and then treated by 25 μmol/L a*t*RAL and/or 80 μmol/L lipophenol for 4 hours. Fluorescence intensity was measured immediately by fluorescence assay in 96‐well plates using CLARIOstar^®^ microplate reader (BMG LABTECH). All experiments were done in quintuple and repeated four times independently.

### 2.9 Catalase activity

ARPE‐19 cells were seeded in six‐well plates and treated as aforementioned. Immediately after treatment, cells were scrapped on ice, centrifuged 5 minutes at 400 *g* and suspended in 100 µL of PBS. For lysis, cells were sonicated 3 × 15 seconds, centrifuged 5 minutes at 14 000 *g* at 4°C and kept on ice. A solution of 0.037% of H_2_O_2_ was prepared. In a spectrophotometer cuvette, 50 µL of sample was added to 1.45 mL of 0.037% H_2_O_2_ and reading of an OD at 240 nm every 20 seconds for 15 minutes to determine the quantity of degraded H_2_O_2_. Data were expressed in nmol of H_2_O_2_ degraded/min/mg of protein.

### 2.10 GSH/GSSG assay

The levels of GSH and GSSG were measured in ARPE‐19 cells plated on tissue culture‐treated white‐with‐clear‐bottom 96‐well plates using the GSH/GSSG‐Glo™ Assay Kit (Promega V6611) according to the manufacturer's instructions. All experiments were performed in triplicate and repeated three times independently.

### 2.11 Western blot analysis

ARPE‐19 cells were lysed in RIPA buffer containing protease inhibitors, homogenized and then centrifuged at 9600 *g* for 3 minutes. Twenty‐five micrograms of the protein lysates in Laemmli buffer were separated on 10% SDS‐PAGE (Mini‐PROTEAN^®^ TGX™ gels, Bio‐Rad) and electrotransferred to PVDF membranes (Trans‐Blot^®^ Turbo™ Transfer System, Bio‐Rad). After blocking, membranes were blotted overnight at 4°C with primary antibodies. After incubation with the corresponding HRP‐conjugated secondary antibodies, detection was performed using an enhanced chemiluminescence kit (Pierce ECL, Thermo Scientific) and recorded by the V3 Western Worflow™ system (Bio‐Rad). The bands were semi‐quantified using densitometry by ImageJ software. Commercial antibodies were used to assess protein expression as follows: monoclonal mouse anti‐GAPDH (Sigma‐Aldrich^®^, G8795 diluted to 1:5000, 37 kD); mouse anti‐α‐tubulin (Sigma‐Aldrich^®^ T5168, diluted to 1:4000, 55 kD); rabbit anti‐cleaved Caspase 3 (Cell Signaling TECHNOLOGY 9661, diluted to 1:200, 17‐19 kD); VDAC (Abcam ab14734, diluted to 1:2500, 32 kD); monoclonal rabbit anti‐catalase (Cell Signaling TECHNOLOGY 12980, diluted at 1:1000, 60 kD); and monoclonal mouse anti‐NQO1 (Cell Signaling TECHNOLOGY 3187, diluted to 1:1000, 29 kD).

### 2.12 Immunofluorescence studies

Mouse NR primary cells or ARPE‐19 cells were fixed in 4% paraformaldehyde for 10 minutes at room temperature (RT), permeabilized in 0.1% triton for 10 minutes at RT, saturated 20 minutes in 0.1% SDS + 10% donkey serum in PBS for 20 minutes at RT and incubated overnight at 4°C with either mouse monoclonal anti‐Rhodopsin antibody RET‐P1 (Novus Biologicals^®^, NBP120‐3267 diluted to 1:500) or rabbit monoclonal anti‐Nrf2 antibody (clone D1Z9C, ref: 12721; Cell Signaling TECHNOLOGY^®^ diluted at 1:200), and detected with Alexa594‐conjugated anti‐rabbit or Alexa488‐conjugated anti‐rabbit secondary antibodies, respectively. Confocal imaging of Rhodopsin and Nrf2 translocation to the nucleus was performed with a Zeiss LSM 5 LIVE DUO Highspeed/ Spectral Confocal system. Image acquisitions were obtained with the Zeiss Zen software.

### 2.13 XCELLigence assay

The xCELLigence system (Roche and ACEA Biosciences) was used to monitor cell adhesion, proliferation and cytotoxicity. The xCELLigence system was connected and tested by Resistor Plate before the RTCA Single Plate station was placed inside the incubator at 37°C and 5% CO_2_. First, the optimal seeding concentration for the proliferation experiments of the ARPE‐19 cells was determined (30 000 cells/well). After seeding of the required number of cells in 100 µL medium per well of a 16‐well E‐plate, the attachment, proliferation and spreading of the cells was monitored every 15 minutes by the xCELLigence system. Approximately 16 hours post‐seeding, when cells were at subconfluency, they were exposed to a*t*RAL 25 μmol/L and/or IP‐DHA 40 μmol/L at the indicated times. Evaluations were performed using xCELLigence 1.2.1 software (Roche and ACEA Biosciences). The real‐time impedance traces were reported using a unitless parameter called Cell Index (CI), where CI = (impedance at time n − impedance in the absence of cells)/nominal impedance value. The experiments were performed using the ARPEGE Pharmacology Screening‐Interactome platform facility at the Institute of Functional Genomics.

### 2.14 Statistics

Statistical analyses were performed using GraphPad Prism 5.0. Software. Data were first analysed with Shapiro‐Wilk normality test, and then, two‐tailed *P*‐values were determined using either the unpaired Student's *t* test or the non‐parametric Mann‐Whitney test. A *P*‐value < .05 was considered significant. The linear correlation was measured by Pearson's *r* correlation coefficient.

## 3 RESULTS

### 3.1 IP‐DHA protects retinal primary cultures against a*t*RAL toxicity

**FIGURE 1:**
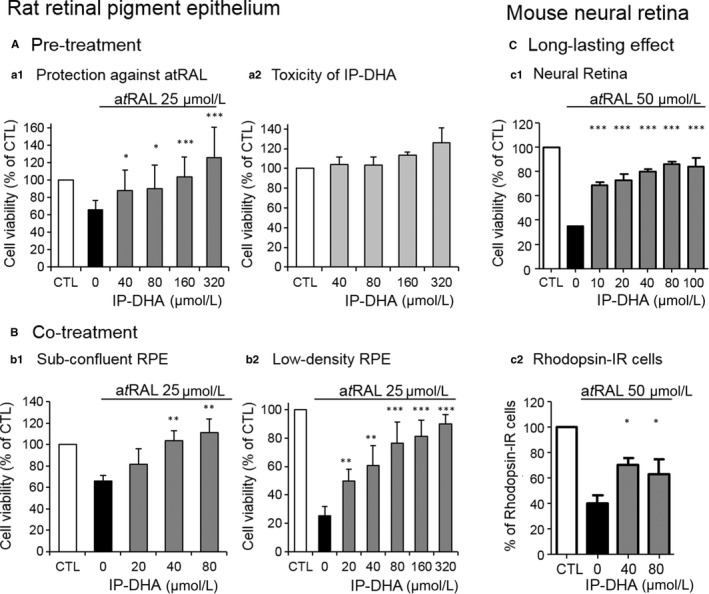
Protection of retinal primary cultures by IP‐DHA against a*t*RAL. A, pre‐treatment of RPE cells with IP‐DHA inhibits a*t*RAL‐induced cell death. A1, rat primary RPE cells were cultured in 96‐well plates and pre‐treated with increasing concentrations of IP‐DHA for 24 h, washed and exposed to 25 μmol/L a*t*RAL for 4 h. A2, RPE cultures were incubated for 24 h with increasing concentrations of IP‐DHA. Cell viability was determined by MTT assay. The data are represented as mean ± SD (n = 7). B, co‐treatment with IP‐DHA and a*t*RAL protects RPE cells. Sub‐confluent (B1) and low‐density (twofold less) (B2) cultures of rat primary RPE cells were cultured in 96‐well plates and co‐incubated with increasing concentrations of IP‐DHA and 25 μmol/L a*t*RAL for 4 h. Cell viability was determined by MTT assay. The data are represented as mean ± SD (n = 3‐6). C, long‐lasting effect of a*t*RAL and IP‐DHA on NR and photoreceptors. NR primary cultures were incubated with increasing concentrations of IP‐DHA for 1 h, and 50 μmol/L a*t*RAL was added for an additional 4 h. The medium was refreshed for the next 20 h. MTT assay measured cell survival (C1) and Rhodopsin‐IR (rhodopsin‐immunoreactivity positive cells) revealed the number of photoreceptor‐derived primary cells (C2). The data are presented as mean ± SEM (n = 3‐4). All data are expressed as a percentage of untreated cells (CTL) *P < .05, **P < .01, ***P < .001 vs a*t*RAL‐treated cells

ABCA4‐associated retinopathies often affect both photoreceptor and RPE in a manner that is not fully elucidated,^5^, ^14^ but which originally involves a defective retinal clearance from the photoreceptor. We tested the protection with IP‐DHA of a*t*RAL‐challenged primary cultures of rat RPE (Figure 1A,B) and mouse NR enriched in photoreceptor cells (Figure 1C). These analyses confirmed significant protective effects of IP‐DHA against a*t*RAL, regardless of the mode of treatment (pre‐ vs co‐treatment Figure 1A,B), the density (Figure 1BB1,BB2), or the cell type (RPE and photoreceptor, Figure 1A,B,C). Furthermore, IP‐DHA did not show cytotoxicity on the primary RPE at a concentration of 320 μmol/L up to eight times higher than the one we previously reported using the ARPE‐19 cell line.^13^ Thus, IP‐DHA is able to protect both RPE and photoreceptor from the toxic effects of a*t*RAL overload without adverse effect.

### 3.2 Structure‐function relationship of IP‐DHA

**FIGURE 2:**
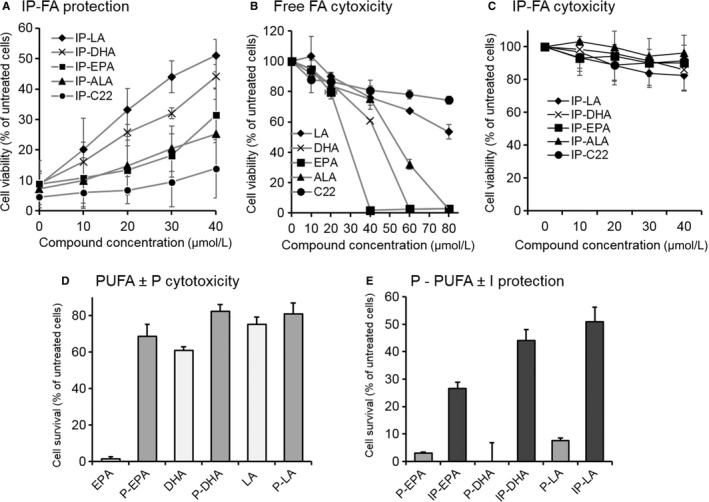
Significance of fatty acid and isopropyl for lipophenol toxicity and protection of ARPE‐19 against a*t*RAL. A, selective protection by IP fatty acid conjugates (IP‐FA). ARPE‐19 cells were cultured in 96‐well plates and incubated with lipophenols (10‐40 μmol/L) for 1 h, and a*t*RAL (25 μmol/L) was added for 4 h co‐incubation. Culture medium was changed, and cell viability was determined 16‐20 h later by MTT assay. Bars indicate SD of the mean (n ≥ 5). Cytotoxicity effects of free fatty acid (B) and of IP‐FA (C). ARPE‐19 cells were incubated with different concentrations for 24 h, and cell viability was determined by MTT assay. PUFA concentrations were selected according to Liu et al^48^ Bars indicate SD of the mean (n = 4‐10). D, E, Significance of phloroglucinol (P) and isopropyl radical (I). Polyunsaturated fatty acid (PUFA) and P‐PUFA conjugates (40 μmol/L) were compared for their cytotoxicity on ARPE‐19 cells for 24 h (D). P‐PUFA (40 μmol/L) was also compared to IP‐PUFA (40 μmol/L) for their efficacies to protect ARPE‐19 from a*t*RAL‐induced cell death (E). Cell viability was determined by MTT assay. Bars indicate SD of the means (n = 3). All data are expressed as a percentage of untreated cells. P, phloroglucinol; IP, isopropyl‐phloroglucinol; LA, linoleic acid (C18:2 ω6); ALA, linolenic acid (C18:3 ω3); EPA, eicosapentaenoic acid (C20:5 ω3); DHA, docosahexaenoic acid (C22:6 ω3); and C22 (saturated)

A selection of fatty acids (omega‐6, omega‐3 and saturated fatty acids) that were conjugated to IP showed a structural selectivity for protection efficacy of ARPE‐19 cells challenged with a toxic dose of a*t*RAL (Figure 2A). The rank of efficacy was LA ≥ DHA > EPA = ALA > C22. This order was not correlated with the level of fatty acid unsaturation (DHA > EPA > ALA > LA > C22), nor with the rank of the toxicity of free fatty acid (EPA > DHA > ALA > LA > C22, Figure 2B). As oxidation levels are correlated with cell toxicity, PUFA toxicity may come from lipid peroxidation.^28^ However, coupling polyunsaturated FA (PUFA) to IP or phloroglucinol (P) significantly reduced PUFA toxicity (Figure 2C,D, respectively). These data highlight that isopropyl does not alter the low toxicity of lipophenols. Regardless of the fatty acid, isopropyl function was necessary for effective protection, the protective effect was lost upon use of non‐alkylated lipophenols (P‐fatty acid, Figure 2E). These results demonstrated that both PUFA and isopropyl are essential for lipophenol activity.

The choice to use IP‐DHA throughout this study rather than IP‐LA, despite the latter's showing better protection against a*t*RAL is justified in view of planned in vivo evaluations. DHA has general and specific transporters to concentrate DHA in the photoreceptors. Therefore, the use of DHA seems more appropriate to improve the uptake of IP‐DHA by the retina. In addition, an omega‐3 such as DHA, which can be released by the enzyme esterase, can have beneficial effects on human retinal diseases that omega‐6, known for their superior pro‐inflammatory properties, cannot reproduce.

### 3.3 Cell‐based assays of IP‐DHA protection

Dynamic cellular biology was first monitored using the xCELLigence System in ARPE‐19 before and after treatment with a*t*RAL and/or IP‐DHA. The system measures electrical impedance which provides quantitative information about the biological status of the cells, including cell number, viability and morphology. The xCELLigence read‐out is a dimensionless parameter called Cell Index (CI) that was normalized with the time‐point before the treatment ~16.5 hours after plating (Figure S1). The addition of 25 μmol/L a*t*RAL significantly decreased the CI, which then stabilized 4 hours later at 11 ± 2% (Figure S1; Table S1). Co‐incubation with 40 μmol/L IP‐DHA limited this decrease to 56 ± 11% two hours after the beginning of treatment, and this effect lasted throughout the analysis (37 hours). We conclude that IP‐DHA responds rapidly against the toxicity of a*t*RAL with persisting protective effect.

**FIGURE 3:**
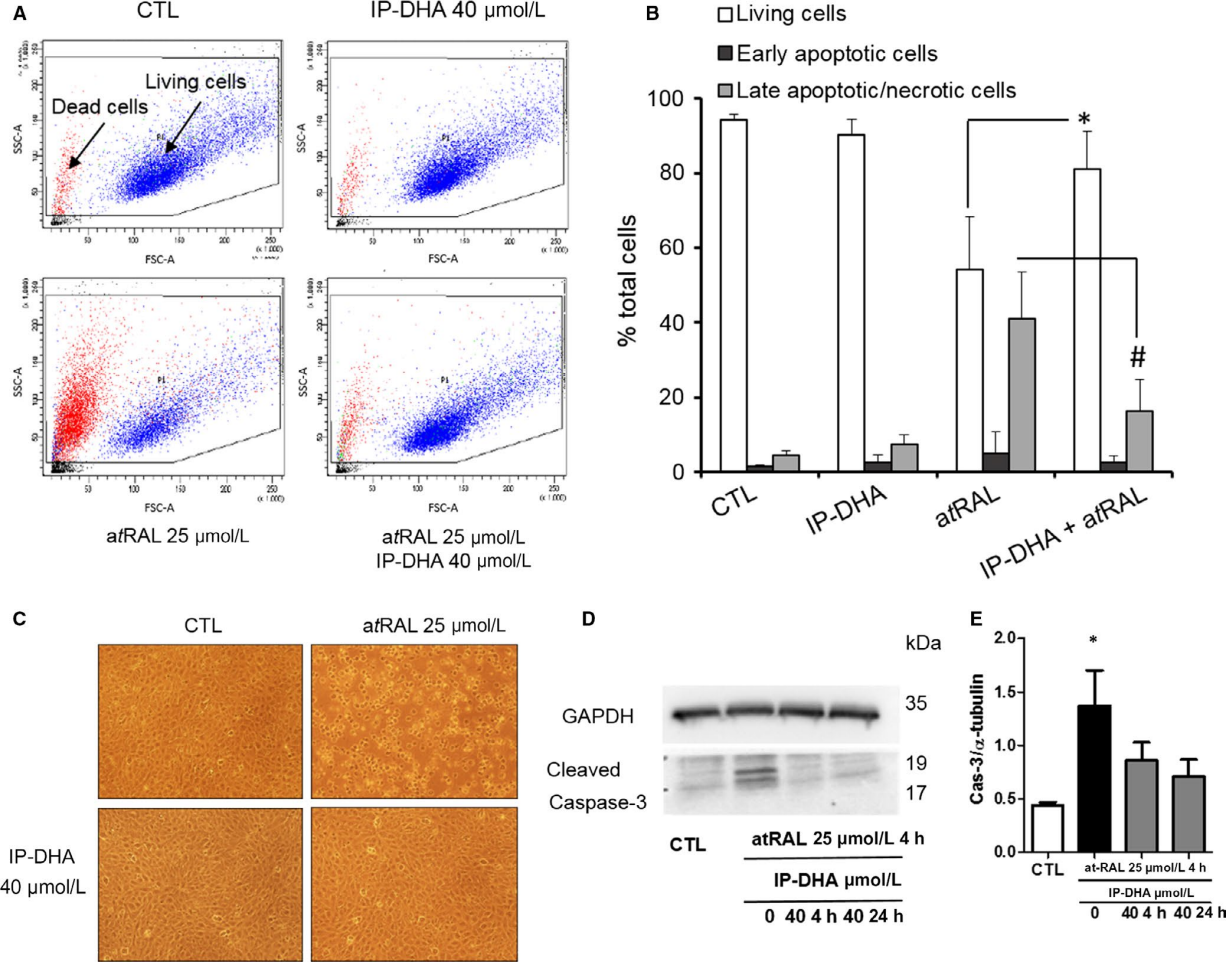
IP‐DHA markedly reduced a*t*RAL‐induced ARPE‐19 cell death. A‐E ARPE‐19 cells were cultured in 24‐well plates and co‐treated for 4 h with 40 μmol/L IP‐DHA and/or 25 μmol/L a*t*RAL. To assess cell death, ARPE‐19 cells were stained with annexin V‐FITC (A) and propidium iodide (PI) and analysed by flow cytometry (A, B). A, living cells are sorted according to their size (FSC) and granularity (SSC). CTL indicates untreated cells. B, the cell populations were identified as follows: living cells (A−, PI−, white squares), early apoptotic cells (A+, PI−, black squares) and late apoptotic/necrotic cells (A+, PI+, grey squares). Results are expressed as a percentage of total cells and presented as mean ± SD (n = 3), C, Photographs show a preservation of ARPE‐19 morphology with 40 μmol/L IP‐DHA even in the presence of 25 μmol/L a*t*RAL. D and E, ARPE‐19 cells showed significantly increased caspase 3 cleavage activity after treatment for 4 h with 25 μmol/L a*t*RAL (n = 3). This activity in IP‐DHA pre‐treated (24 h) and co‐treated (4 h) cells did not show significant changes when compared to each other or with the untreated cells (CTL)

Consequently, we applied a 4‐hour co‐treatment with a*t*RAL and IP‐DHA to assess the protection against detrimental effects induced by a*t*RAL overload in ARPE‐19 (Figure 3). As shown in Figure 3A,B, 94.2 ± 1.7% untreated and 90.1 ± 4.4% IP‐DHA‐treated cells survived respectively, suggesting no adverse effect of IP‐DHA. Twenty‐five μmol/L a*t*RAL reduced cell survival to 54.1 ± 14.2% by apoptosis and 40.9 ± 12.5% by necrosis whereas co‐incubation with 40 μmol/L IP‐DHA significantly increased the living cells to 81.2 ± 9.9%, and reduced necrotic cells to 16.3 ± 8.4%. The absolute values generated by the flow cytometry experiments were higher than those of xCELLigence. The difference between the protocols (toxicity of a*t*RAL) and the measured parameters may be one explanation. It can be assumed that the ARPE‐19 cells lose their adhesion but are still alive. Nevertheless, both cellular tests show a similar trend on the survival capacity of ARPE‐19 cells co‐treated with a*t*RAL and IP‐DHA compared to a*t*RAL.

**FIGURE 4:**
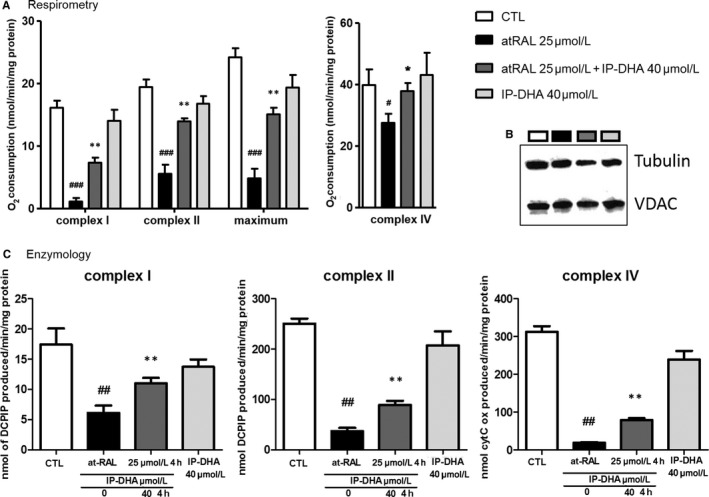
The mitochondrial poisoning by a*t*RAL is partially rescued by IP‐DHA in ARPE‐19. A and B, mitochondrial poisoning by a*t*RAL is partially rescued by IP‐DHA. Respirometry estimates the rates of oxygen consumption which is governed by the mitochondrial respiratory complex chain. The respiration driven by (A) and enzymatic activities of (C) complexes I, II and IV were severely affected by 25 μmol/L a*t*RAL but co‐treatment with IP‐DHA (40 μmol/L) significantly rescues both parameters. Results are expressed as means ± SEM (n ≥ 4). B, mitochondrial mass was not affected by treatment as demonstrated by a constant immunodetection of VDAC (outer mitochondrial membrane ion channel). ^#^ *P* < .05, ^##^ *P* < .01, ^###^ *P* < .001 vs untreated CTL, and *P < .05, **P < .01, ***P < .001 vs a*t*RAL‐treated cells

Morphologic changes in ARPE‐19 cells were observed following a*t*RAL exposure (Figure 3C) and an apoptotic caspase 3‐cleavage signal was detected by Western blot (Figure 3D,E). Long (24 hour's pre‐) and short (4‐hour co‐) IP‐DHA treatment restored healthy morphology and abolished the caspase‐dependent apoptosis. Previous reports revealed that a*t*RAL could directly act on and elicit a poisonous effect in mitochondria.^3^, ^7^ We therefore performed respirometry to assess the functionality of mitochondrial RCC (Figure 4A). Respiration driven by complexes I, II and IV was evaluated. All responses were dramatically diminished by a*t*RAL treatment. IP‐DHA co‐treatment partially but significantly rescued the functionality of the mitochondrial respiratory chain. Notably, the constant expression of VDAC (Figure 4B), a major protein of the outer mitochondrial membrane, suggested that a*t*RAL does not induce loss of the mitochondrial mass. Moreover, direct measurement of enzymatic activities clearly showed that all RCC were impaired by a*t*RAL and partially rescued by IP‐DHA treatment (Figure 4C). Thus, the protective effect of IP‐DHA seems to apply directly to the mitochondrial respiration essential to the cell viability.

### 3.4 Molecular and cellular mechanisms of IP‐DHA protection

**FIGURE 5:**
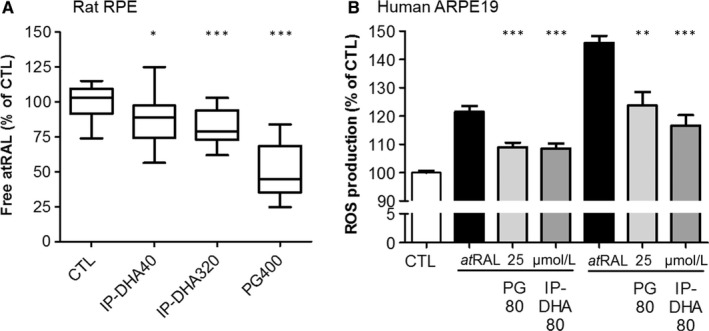
IP‐DHA decreases free a*t*RAL and prevents a*t*RAL‐induced ROS production in RPE. A, Primary rat RPE cells were cultured in 96‐well plates, co‐treated with 25 μmol/L a*t*RAL and 400 μmol/L phloroglucinol (PG) or 40 and 320 μmol/L IP‐DHA for 4 h, and retinoids were extracted from cell lysates. The free a*t*RAL content was quantified by HPLC from standard calibration. The data are from a representative experiment performed in triplicate and represented as mean ± SD (n = 3). The data are expressed as a percentage of untreated cells (CTL). B, ARPE‐19 cells were cultured in 96‐well plates, pre‐treated with 80 μmol/L phloroglucinol (PG) or 80 μmol/L IP‐DHA for 24 h before incubation with H_2_DCFDA for 30 min, and then, a*t*RAL (25 or 50 μmol/L) was added for an additional 4 h. Fluorescent intensity was measured at λem 535 nm (λexc = 485 nm) and expressed as a percentage of untreated cells (CTL). The data are expressed as mean ± SEM (n = 4). *P < .05, **P < .01, ***P < .001 vs a*t*RAL‐treated cells

Natural polyphenols were reported as potent against COS involved in age‐related diseases,^29^ either as sequestrating agents of reactive aldehydes and scavengers of reactive species, or by the activation of Nrf2 transcription factor that promotes expression of many phase II detoxifying enzymes.^30^ We recently showed that phloroglucinol acts as an anti‐COS agent trapping a*t*RAL and scavenging ROS produced by H_2_O_2_ treatment (identified by DCFDA probes.^9^, ^27^ Here, we consider the anti‐COS capacity of IP‐DHA compared to phloroglucinol in RPE cells (Figure 5). IP‐DHA reduced both free a*t*RAL and ROS produced by a*t*RAL treatment (Figure 5A and 5B, respectively). A*t*RAL treatment is able to alter the respiratory chain in mitochondria and potentially disrupts homeostasis in the ER, thereby initiating ER stress, which in turn induces ROS generation such as O_2_^−·^and then HO^·^.^31^ IP‐DHA was effective in reducing the concentration of free a*t*RAL (Figure 5A) at doses that provided partial (40 μmol/L IP‐DHA, 87.0 ± 4.4% of control free atRAL) and total (320 μmol/L IP‐DHA, 81.6 ± 3.7% of control free a*t*RAL) protection of primary rat RPE challenged with 25 μmol/L a*t*RAL. As we previously described,^9^ 400 μmol/L (50 µg/mL) phloroglucinol was most efficient (Figure 5A, 51.3 ± 4.6% of control free a*t*RAL). On the other hand, the two compounds were equipotent for decreasing a*t*RAL‐mediated ROS production (Figure 5B). Together, our observations suggest that reducing excess a*t*RAL and oxidative stress status may account for the protective effect of lipophenols on RPE cells.

**FIGURE 6:**
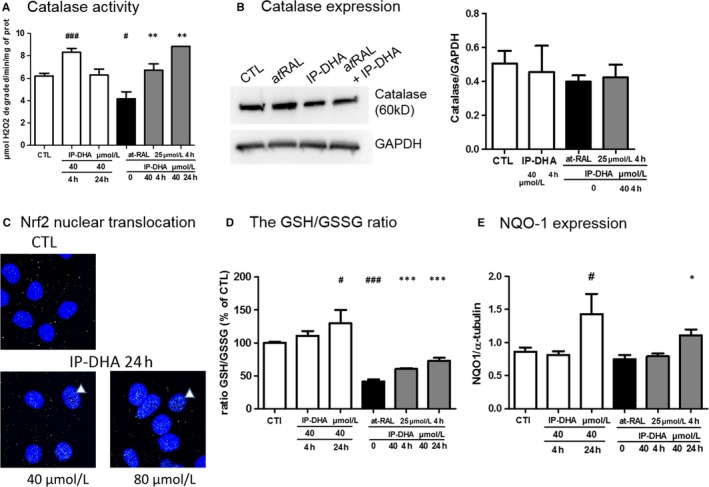
Effect of IP‐DHA on antioxidant defence enzymes in ARPE‐19. Effect of IP‐DHA on antioxidant enzymes. Catalase activity (A) and its expression (B). ARPE‐19 cells were incubated in 6‐well plates for 4 h or 24 h with 40 μmol/L IP‐DHA with or without 25 μmol/L a*t*RAL during the last 4 h. Catalase activity was measured in cell lysates and expressed in μmol of H_2_O_2_ degraded/min/mg of protein. Catalase expression was quantified by Western blot analysis with a monoclonal rabbit anti‐catalase antibody and enhanced chemiluminescence (ECL) detection using densitometry and ImageJ software. GAPDH expression was used as a loading control. Results are expressed as mean ± SEM (n ≥ 3). C, Nrf2 expression and nuclear translocation were explored by immunofluorescence with a rabbit monoclonal anti‐Nrf2 antibody and Alexa488‐conjugated anti‐rabbit. Nuclei were stained with the blue fluorescent Hoechst dye. Confocal imaging revealed increased green spots in the nuclei after 24‐h treatment with IP‐DHA. Similar ARPE‐19 treatments were performed, and GSH/GGSSG ratio (D) and NQO‐1 expression (E) were quantified. Results are expressed as mean ± SEM (n = 4). ^#^ *P* < .05, ^###^ *P* < .001 vs untreated CTL and *P < .05, **P < .01, ***P < .001 vs a*t*RAL‐treated cells

Besides their scavenging potency, anti‐COS compounds also were described as inducers of phase II antioxidant and detoxifying enzymes, which contribute efficiently to the redox homeostasis and prevent retinal cell death both in vivo and in vitro.^30^, ^32^ Activation of the EpRE enzymes through Nrf2/Keap1 pathway activation is one of the mechanism of polyphenol antioxidant activity. We measured the activity and expression of key enzymes in defence of the retina, including catalase, glutathione related enzymes and NAD (P) H quinine oxidoreductase (NQO‐1; Figure 6). A 4‐hour treatment with IP‐DHA increased the catalase activity and prevented its decrease by a*t*RAL (Figure 6A), whereas it did not regulate its expression (Figure 6B). The 24 hours pre‐treatment with IP‐DHA increased in a dose‐dependent manner the expression of Nrf2 and its nuclear translocation in ARPE‐19 cells (Figure 6C). The same treatment increased the GSH/GSSG ratio (Figure 6D) and the expression of NQO‐1 (Figure 6E), suggesting an up‐regulation of redox regulating and detoxifying enzymes. These results support the notion that the protective role of IP‐DHA in retinal cells involves both molecular (a*t*RAL reduction) and cellular (enzymatic) mechanisms.

### 3.5 IP‐DHA rescues cells under toxic effect of blue light‐exposed A2E

**FIGURE 7:**
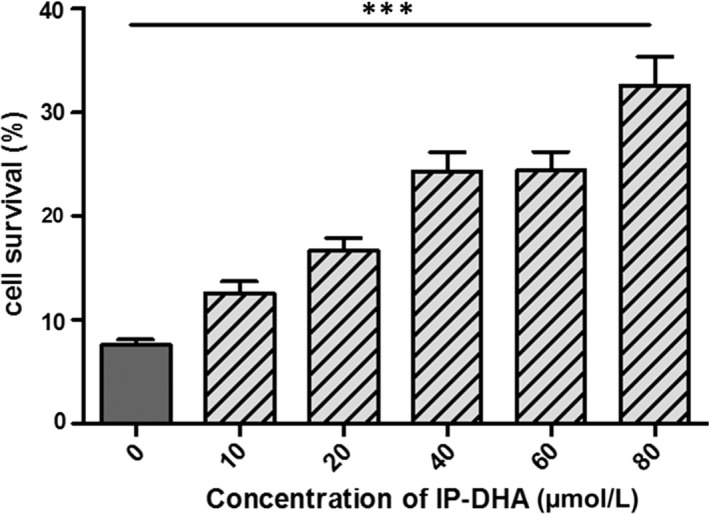
Protecting effect of IP‐DHA against photo‐oxidized A2E in ARPE‐19. ARPE‐19 cells were plated in 96‐well plates (4 × 10^4^ cells/well) and grown until confluency. Then, cells were untreated (grey bar) or treated with increasing doses of IP‐DHA (hatched bars) for 1 h, prior to incubation with A2E (20 μmol/L) for 6 h. Cells were exposed to blue light (λmax 430‐470 nm, 4600 lux) and grown for 24 h before cell survival was determined by MTT assay. Data are expressed as a percentage of control untreated cells not exposed to A2E) and are presented as mean ± SD (n = 3 independent experiments). Unpaired Student's *t* test, ***P < .001, non‐treated vs exposed cells to photo‐oxidized A2E. The maximum absorption of IP‐DHA was 218 and 272 nm (see UV spectrum of IP‐DHA in Supporting Information) which differs from that of blue light excitation (430‐470 nm) and A2E maximum absorption (337‐437 nm)

The daily shedding of the distal tips of the outer segment followed by their phagocytosis in RPE cells leads to accumulation of bisretinoids in lysosomes and formation of A2E.^33^ A2E is a pigment which captures blue light and produces reactive carbonyl and oxygen species that can lead to cell death. We tested whether IP‐DHA may inhibit the photo‐induced toxic effect of A2E in ARPE‐19 cells (Figure 7). The latter were incubated with 20 μmol/L pure synthetic A2E for 6 hours following the addition of IP‐DHA at various concentrations for 1 hour. Medium was replaced, and cells were exposed to blue light (430‐470 nm, 4600 lux) for 30 minutes before they were returned to the incubator at 37°C overnight. Cell survival analysis using a MTT assay showed that IP‐DHA dose‐dependently recovers survival from A2E‐treated cells. At 80 μmol/L, IP‐DHA treatment markedly increases cell survival by fourfold.

## 4 DISCUSSION

The pathophysiological mechanisms of STGD1, first involve alterations in the photoreceptors due to mutations in the ATP‐binding cassette transporter *ABCA4* gene, delay in a*t*RAL reduction, and accumulation of autofluorescent bisretinoids in photoreceptors by condensation of a*t*RAL and phosphatidylethanolamine.^34^ At this stage, a*t*RAL reactivity is responsible for COS.^9^, ^13^ Later, phagocytosis transfers bisretinoid‐burdened POS to the RPE where bisretinoids can account for autofluorescence of lipofuscin, light‐dependent COS and consequently death of RPE.^33^ Therefore, COS play a crucial role throughout the disease from its onset in the photoreceptors to its progression in the RPE. Thus, it is highly relevant to develop new therapeutic compounds capable of limiting COS in the outer retina.

Polyphenols have long been recognized as antioxidant and more recently as anti‐carbonyl stress derivatives, and their application in the treatment of neurodegenerative diseases has been widely acknowledged in the past few years.^35^, ^36^ Among them, phloroglucinol is a monomer of phlorotannins, which also displays therapeutic potential for neurodegenerative diseases.^37^, ^38^ Neurodegeneration is a multifactorial process and polyphenols present pleiotropic effects (antioxidant, anti‐inflammatory, immunomodulatory properties) due to their ability to modulate the activity of multiple targets involved in pathogenesis, thereby halting the progression of these diseases. We previously reported cytoprotective effects of phloroglucinol in outer retinal cells by scavenging ROS and trapping a*t*RAL.^9^ However, a major disadvantage of phloroglucinol is its low bioavailability in the retina (unpublished personal data). Our strategy to improve selectivity for the retina relied on chemical modifications of the resorcinol core. We synthesized phloroglucinol derivatives by attaching DHA on a phenolic group. The choice of DHA was dictated by its high content in the photoreceptor disc membrane, the site of photoisomerization where a*t*RAL is produced. Moreover, DHA has several advantages in the retina (a) it is avidly uptaken by RPE and retained in the POS,^39^, ^40^ (b) it is essential for preserving visual functions and maintaining disc properties in the POS,^41^ (c) it facilitates the clearance of free retinal to prevent the accumulation of bisretinoid compounds associated within macular disease,^42^ and (d) it is a precursor of neuroprotectin D1 which protects the retina against oxidative stress induced by cell‐injury‐induced.^43^ The second modification to phloroglucinol was the introduction of an isopropyl radical, whose electron‐donating inductive effect should adjust the nucleophilicity of the aromatic ring to trap a*t*RAL most efficiently.

Then, we evaluated the protective effect of IP‐DHA against a*t*RAL toxicity in outer retinal cells. IP‐DHA was shown to be effective both in RPE and in NR. In the RPE, we showed that IP‐DHA protects very well against a*t*RAL compared to other lipophenols tested. IP‐DHA and IP‐LA are the most effective although at a different degree of unsaturation of PUFA. IP‐C22 with a saturated C22 lipid chain has a very low efficiency comparable to that of phloroglucinol,^13^ showing the need for unsaturation in the fatty acid moiety. An explanation for this is an improvement in lipophilicity and an enhancement in cell permeability. We have shown in this study that the protective effect appears on the first hours of treatment and persists overtime, suggesting that lipophenol can be rapidly available and stabilized into the cell. The present data demonstrate that the PUFA grafting on alkylated phloroglucinol promotes survival of RPE cells.

Hence, we tried to elucidate further the mechanism of action of IP‐DHA and compared its efficacy to that of phloroglucinol. Firstly, IP‐DHA, but also phloroglucinol (albeit, at high concentration), efficiently reduces a*t*RAL in primary cultures of rat RPE. Moreover, the production of ROS induced by a*t*RAL and identified by H_2_DCFDA is almost equally reduced by IP‐DHA or by phloroglucinol. Together, these data suggest that IP‐DHA and phloroglucinol are equally effective as anti‐COS. However, as shown in our previous work,^13^ protection of ARPE‐19 cells from a*t*RAL toxicity by IP‐DHA is four times greater than by phloroglucinol itself. We hypothesize that this could be due to: (a) a better bioavailability of IP‐DHA due to high lipophilicity of DHA, and/or (b) the DHA fatty acyl chain that can promote the “direct reaction” (nucleoplilic addition) of IP‐DHA onto a*t*RAL by lowering the activation energy required to scavenge a*t*RAL and/or (c) IP‐DHA triggering indirect cell defence enzyme systems. Cellular adducts between IP‐DHA and a*t*RAL have not yet been identified in vitro (chemical reaction and ARPE‐19 incubation). By contrast, we were able to highlight the formation of retinal adducts with IP as a mixture of chromene‐isopropyl (data not shown). We concluded that it was unlikely the presence of DHA would facilitate the reaction of IP‐DHA on a*t*RAL. The hypothesis that DHA improves cell bioavailability should be retained but requires further investigation by tracking IP‐DHA into the cell. Interestingly, IP‐DHA and IP‐LA could be targeted rather than mitochondria where they could prevent a*t*RAL from provoking ROS generation and COS in the RPE, thus protecting cells from apoptosis.^3^, ^44^ The exact mechanisms of protection against a*t*RAL toxicity are still unclear. The electron‐donating inductive isopropyl ether group on phloroglucinol is necessary to increase its ability (electron enrichment of the aromatic ring) to protect against reactive electrophile a*t*RAL.^13^ However, an additional role of the isopropyl function is not excluded, such as the steric hindrance in the vicinity of the DHA ester function that could protect it from esterase and improve the stability of the lipophenol. This latter point could not be established in this work, and it is not clear at present whether it could modulate the reactivity of phloroglucinol on a*t*RAL. Further study is needed for elucidating the role of those two functions in more detail. Regarding reduction of oxidative status, we show that IP‐DHA is able to up‐regulate enzymatic responses in the short, as well as, in the long‐term, by increasing the constitutive (catalase) or the inducible (Nrf_2_‐dependent Electrophilic Responsive Element (EpRE) pathway) enzymatic antioxidant activities, respectively. Thus, mechanism of action of IP‐DHA may pass through direct ROS scavenging and/or by stimulating the production of several detoxifying enzymes of the EpRE through the transcription activity of Nrf2. Phloroglucinol itself has been described as being able to play a role in the antioxidant defences, either directly (radical scavenging of ROS) or, more efficiently, by activating the Nrf2 pathway, in the case of a low level of oxidative stress.^37^ With these latest results, it is possible to summarize the optimal characteristics of the chemicals showing the highest efficacy against STGD1, as well as the proven or most likely underlying mechanisms of action (table S2).

In the prospective treatment of patients with IP‐DHA, we assume that the release of free DHA and IP may be part of the mechanism of action of the compound, as the ester bond could be cleaved by a plasma and/or cellular esterase. This is not a drawback as many studies show the beneficial effects of DHA supplementation in AMD and STGD1^45^, ^46^ and dietary polyphenols in AMD against oxidative stress and beyond.^47^ In addition, the oxidation of DHA not only causes deleterious effects (lipid peroxidation), but should also contribute to the release of cellular mediators (neuroprostane, neuroprotectin D1) helping the cell to fight oxidation. In addition, the antioxidant activity of phloroglucinol would enhance the beneficial effect on vision.

In conclusion, our data show that IP‐DHA is effective to protect outer retinal cells against lethal dose of a*t*RAL. The beneficial role of IP‐DHA in retinal cells involves both anti‐carbonyl and anti‐oxidative capacities. This suggests potential effects of lipophenols in the prevention of macular degeneration associated with COS, such as STGD1 and AMD. Additional studies will be necessary to examine the effect of IP‐DHA in animal models of macular degeneration.

## CONFLICT OF INTEREST

The authors have declared that no conflict of interest exists.

## AUTHORS CONTRIBUTIONS

AC, EM, LG, NJ, MR and CA‐P. conducted the experiments; AC, DC, CC and PB designed the research studies; AC, EM, DC, CA‐P. and PB acquired and analysed data; PB prepared the published work and wrote the initial draft; DC, CC and JV reviewed and improved the manuscript; GL, TD and PB acquired financial support for the project; JV, CC and PB supervised the research activity; and IM coordinated the Centre for Inherited Sensory Disorders.

## Supporting information

Supplementary Material

## Data Availability

The data that support the findings of this study are available from the corresponding author upon reasonable request.
