# Supplementary material for: Isopropyl‐phloroglucinol‐DHA protects outer retinal cells against lethal dose of all‐trans‐retinal
Source: J Cell Mol Med. 2020 Mar 25;24(9):5057–69. doi: 10.1111/jcmm.15135 (PMC7205824; doi:10.1111/jcmm.15135)
Supplement: Supplementary file 1 — Supplementary Material [file JCMM-24-5057-s001.docx]

**Supporting Information**

Chemical synthesis of lipophenols

**General Procedure for coupling step between Fatty acid and polyphenolic derivatives:** DHA (1.1 equiv., 0.30 mmol) and each of the concerned phenolic derivatives (1 equiv., 0.27 mmol) were dissolved in dry CH_2_Cl_2_ (6 mL). DCC (1.1 equiv., 0.30 mmol) and DMAP (0.1 equiv, 0.03 mmol) were added to the solution and the reaction was stirred at room temperature for 5h under nitrogen. The mixture was left 2h at 4°C to induce dicyclohexylurea crystallization. The urea residue was then filtered off, and the filtrate was washed with water and brine. The organic layer was dried on MgSO_4_ and concentrated under reduced pressure. Purification of the crude material was performed by chromatography on silica gel to afford the desired lipophenol.

**General Procedure for deprotection of TIPS protecting group on Fatty acid-polyphenol derivatives:** To a solution of the appropriate protected DHA-polyphenol (1 equiv., 0.19 mmol) in anhydrous THF (13 mL), was added dropwise triethylammonium trihydrofluoride (Et_3_N-3HF, 3 equiv., 0.57 mmol for mono-protected compounds or 6 equiv., 1.14 mmol for di-protected derivatives). The reaction was stirred at room temperature during 4h to 6h, until completion of the reaction. AcOEt (40 mL) was added to the mixture and the organic layer was washed with water (15 mL) and brine (15 mL). The organic phase was dried (MgSO_4_) and concentrated under vacuum. The residue obtained was purified by chromatography on silica gel to give the deprotected lipophenol.

**Synthesis of Phloroglucinol-DHA conjugate (P-DHA) and Phloroglucinol -isopropyl-DHA conjugate (IP-DHA) was performed as described in [**[**13**](#_ENREF_13)**]**

**Synthesis of alkylated Phloroglucinol- EPA conjugate (IP-EPA)**

**(5,8,11,14,17 Z)-3-hydroxy-5-isopropoxyphenyl icosa-5,8,11,14,17-pentaenoate**

Coupling of the mono-TIPS-mono-isopropyl-phloroglucinol [[13](#_ENREF_13)] (70 mg, 0.22 mmol) and EPA ( 65 mg, 0.22 mmol) was performed according to the general procedure and afforded the protected mono-isopropyl-phloroglucinol-EPA (71 mg, 54 %) as an uncoloured oil after purification on silica gel chromatography (hexane/AcOEt 99.5/0.5).

^1^H NMR (500 MHz, CDCl_3_) δ = 6.28 (t, *J*=2.2, 1H), 6.23 (t, *J*=2.1, 1H), 6.20 (t, *J*=2.1, 1H), 5.47 – 5.27 (m, 10H), 4.44 (hept, *J*=6.1, 1H), 2.93 – 2.75 (m, 8H), 2.57 – 2.45 (m, 2H), 2.25 – 2.14 (m, 2H), 2.13 – 2.01 (m, 2H), 1.81 (q, *J*=7.5, 2H), 1.31 (d, *J*=6.1, 6H), 1.28 – 1.18 (m, 3H), 1.09 (d, *J*=7.3, 18H), 0.97 (t, *J*=7.5, 3H); ^13^C NMR (126 MHz, CDCl_3_) δ 172.0, 159.5, 157.7, 152.3, 132.4, 129.4, 129.2, 128.9, 128.6, 128.6, 128.5, 128.4, 128.2, 127.3, 106.3, 105.7, 102.6, 70.5, 34.1, 26.9, 26.0, 26.0, 26.0, 25.9, 25.0, 22.3(2C), 20.9, 18.2 (6C), 14.6, 12.9(3C).

Deprotection of this protected EPA-phloroglucinol (65 mg, 0.11 mmol) was performed using the general procedure and afforded (5,8,11,14,17 Z)-3-hydroxy-5-isopropoxyphenyl icosa-5,8,11,14,17-pentaenoate (37 mg, 76%) as an uncoloured oil after purification on silica gel chromatography (hexane/AcOEt 9/1).

R_f_ (hexane/AcOEt 9/1) 0.3; ^1^H NMR (500 MHz, CDCl_3_) δ 6.22 (t, *J* = 2.2 Hz, 1H), 6.18 (t, *J* = 2.1 Hz, 1H), 6.14 (t, *J* = 2.1 Hz, 1H), 5.64 (br, 1H), 5.48 – 5.28 (m, 10H), 4.44 (quint, *J* = 6.0 Hz, 1H), 2.83 – 2.80 (m, 8H), 2.56- 2.53 (m, 2H), 2.21-2.17 (m, 2H), 2.12 – 2.02 (m, 2H), 1.82 (quint, *J* = 7.5 Hz, 2H), 1.30 (d, *J* = 6.0 Hz, 6H), 0.97 (t, *J* = 7.5 Hz, 3H); ^13^C NMR (126 MHz, CDCl_3_) δ 172.7, 159.8, 157.5, 152.3, 132.4, 129.4, 129.0, 128.9, 128.6, 128.5, 128.5, 128.4, 128.2, 127.3, 101.9, 101.9, 101.3, 70.6, 34.0, 26.8, 25.9, 25.9, 25.8, 25.8, 25.0, 22.2, 20.9(2C), 14.6.

**Preparation of (9,12,15Z)-3-hydroxy-5-isopropoxyphenyl octadeca-9,12,15-trienoate (P-OiP-ALA)**

**(9,12,15Z)-3-isopropoxy-5-((triisopropylsilyl)oxy)phenyl octadeca-9,12,15-trienoate**

Coupling of the mono-TIPS-mono-isopropyl-phloroglucinol [[13](#_ENREF_13)] (70 mg, 0.22 mmol) and ALA (60 mg, 0.22 mmol) was performed according to the general procedure and afforded the protected mono-isopropyl-phloroglucinol-ALA (101 mg, 80 %) as an uncolored oil after purification on silica gel chromatography (hexane/AcOEt 99.5/0.5).

Rf (hexane/AcOEt 99/1) 0.2; 1H NMR (500 MHz, CDCl3) δ 6.28 (t, J = 2.1 Hz, 1H), 6.23 (t, J = 2.1 Hz, 1H), 6.20 (t, J = 2.1 Hz, 1H), 5.42 – 5.29 (m, 6H), 4.44 (sept, J = 6.0 Hz, 1H), 2.82 – 2.80 (m, 4H), 2.50 (t, J = 7.5 Hz, 2H), 2.11-2.04 (m, 4H), 1.72 (quint, J = 7.5 Hz, 2H), 1.43-1.34 (m, 8H), 1.30 (d, J = 6.0 Hz, 6H),1.27-1.19 (m, 3H), 1.09 (d, J = 7.3 Hz, 18H), 0.97 (t, J = 7.5 Hz, 3H); 13C NMR (126 MHz, CDCl3) δ 172.3, 159.5, 157.7, 152.3, 132.3, 130.6, 128.6, 128.6, 128.1, 127.4, 106.4, 105.6, 102.7, 70.5, 34.8, 29.9, 29.5, 29.5, 29.4, 27.5, 25.9, 25.9, 25.2, 22.3(2C), 20.9, 18.2(6C), 14.6, 12.9 (3C).

Deprotection of this protected phloroglucinol-ALA (90 mg, 0.15 mmol) was performed using the general procedure and afforded compound P-OiP-ALA (56 mg, 58%) as an uncolored oil after purification on silica gel chromatography (hexane/AcOEt 9/1).

Rf (hexane/AcOEt 8/2) 0.5; 1H NMR (500 MHz, CDCl3) δ 6.22 (t, J = 2.1 Hz, 1H), 6.17 (t, J = 2.1 Hz, 1H), 6.13 (t, J = 2.1 Hz, 1H), 5.87 (Br, 1H), 5.42 – 5.29 (m, 6H), 4.44 (sept, J = 6.0 Hz, 1H), 2.82 – 2.80 (m, 4H), 2.52 (t, J = 7.5 Hz, 2H), 2.11-2.04 (m, 4H), 1.73 (quint, J = 7.5 Hz, 2H), 1.43-1.32 (m, 8H), 1.30 (d, J = 6.0 Hz, 6H), 0.97 (t, J = 7.5 Hz, 3H); 13C NMR (126 MHz, CDCl3) δ 173.1, 159.8, 157.6, 152.3, 132.3, 130.6, 128.6, 128.6, 128.1, 127.4, 102.0, 101.9, 101.3, 70.6, 34.8, 29.9, 29.5, 29.4, 29.4, 27.5, 25.9, 25.8, 25.2, 22.2, 20.9(2C), 14.6.

**Preparation of (9,12Z)-3-hydroxy-5-isopropoxyphenyl octadeca-9,12-dienoate (P-OiP-LA)**

**(9,12Z)-3-isopropoxy-5-((triisopropylsilyl)oxy)phenyl octadeca-9,12-dienoate**

Coupling of the mono-TIPS-mono-isopropyl-phloroglucinol {Crauste, 2014 #237;Crauste, 2014 #237}[[13](#_ENREF_13)] (70 mg, 0.22 mmol) and LA (60 mg, 0.22 mmol) was performed according to the general procedure and afforded the protected mono-isopropyl- phloroglucinol-LA (106 mg, 83 %) as an uncolored oil after purification on silica gel chromatography (hexane/AcOEt 99.5/0.5).

Rf (hexane/AcOEt 99/1) 0.1; 1H NMR (500 MHz, CDCl3) δ 6.28 (t, J = 2.5 Hz, 1H), 6.23 (t, J = 2 Hz, 1H), 6.20 (t, J = 2 Hz, 1H), 5.41 – 5.29 (m, 4H), 4.44 (sept, J = 6.0 Hz, 1H), 2.78 – 2.76 (m, 2H), 2.50 (t, J = 7.5 Hz, 2H), 2.07-2.03 (m, 4H), 1.72 (quint, J = 7.5 Hz, 2H), 1.43-1.28 (m, 14H), 1.31 (d, J = 6.0 Hz, 6H),1.27-1.20 (m, 3H), 1.09 (d, J = 7.5 Hz, 18H), 0.89 (t, J = 7 Hz, 3H); 13C NMR (126 MHz, CDCl3) δ 172.3, 159.4, 157.7, 152.3, 130.6, 130.3, 128.4, 128.2, 106.4, 105.6, 102.7, 70.5, 34.8, 31.9, 29.9, 29.7, 29.5, 29.5, 29.4, 27.5, 27.5, 25.9, 25.2, 22.9, 22.3(2C), 18.2(6C), 14.6, 12.9(3C).

Deprotection of the protected LA-IP-phloroglucinol (100 mg, 0.17 mmol) was performed using the general procedure used in example 1 and afforded coumpound P-OiP-LA (48 mg, 65%) as an uncolored oil after purification on silica gel chromatography (hexane/AcOEt 9/1).

Rf (hexane/AcOEt 8/2) 0.5; 1H NMR (500 MHz, CDCl3) δ 6.21 (t, J = 2 Hz, 1H), 6.17 (t, J = 2 Hz, 1H), 6.13 (t, J = 2 Hz, 1H), 5.90 (br, 1H), 5.39 – 5.34 (m, 4H), 4.44 (sept, J = 6.0 Hz, 1H), 2.79 – 2.76 (m, 2H), 2.52 (t, J = 7.5 Hz, 2H), 2.08-2.03 (m, 4H), 1.73 (quint, J = 7.5 Hz, 2H), 1.39 – 1.25 (m, 14H), 1.29 (d, J = 6.0 Hz, 6H), 0.89 (t, J = 7 Hz, 3H); 13C NMR (126 MHz, CDCl3) δ 173.1, 159.8, 157.6, 152.3, 130.5, 130.3, 128.4, 128.2, 102.0, 101.9, 101.3, 70.6, 34.8, 31.8, 29.9, 29.7, 29.5, 29.4, 29.4, 27.5, 27.5, 25.9, 25.2, 22.9, 22.2(2C), 14.4.

**Synthesis of alkylated Docosanoic-Phloroglucinol conjugate (P-OiP-C22)**

**3-hydroxy-5-isopropoxyphenyl docosanoate**

Docosanoic acid (136.6 mg, 0.40 mmol) and the mono-TIPS-mono-isopropyl-phloroglucinol [[13](#_ENREF_13)] (100 mg, 0.30 mmol) were dissolved in dry CH_2_Cl_2_ (6 mL) and dry DMF (1.5 mL). DCC (82.7, 0.40 mmol) and DMAP (5 mg, 0.04 mmol) were added to the solution and the reaction was stirred at room temperature for 5h under nitrogen, and then overnight at 50°C. Then, the mixture was left 2h at 4°C to induce dicyclohexylurea crystallization. The urea residue was then filtered off, and the filtrate was washed with water and brine. The organic layer was dried on MgSO_4_ and concentrated under reduced pressure. Purification of the crude material was performed by chromatography on silica gel (hexane/AcOEt 99.5/0.5) to afford (95 mg, 48%) the protected derivative as an uncoloured oil.

^1^H NMR (500 MHz, CDCl_3_) δ = 6.29 (t, *J*=2.2, 1H), 6.24 (t, *J*=2.1, 1H), 6.21 (t, *J*=2.1, 1H), 4.45 (hept, *J*=6.0, 1H), 2.51 (t, *J*=7.5, 2H), 1.73 (q, 7.5, 2H), 1.43 – 1.18 (m, 42H), 1.10 (d, *J*=7.3, 18H), 0.88 (t, *J*=7.0, 3H); ^13^C NMR (126 MHz, CDCl_3_) δ = 172.3, 159.5, 157.7, 152.4, 106.8, 105.6, 102.7, 70.4, 34.8, 32.3, 30.1, 30.0(8C), 30.0, 30.0(2C), 29.8, 29.7, 29.6, 29.4, 25.3, 23.0, 22.3(2C), 18.2(6C), 14.5, 12.9(3C).

Deprotection of this protected-docosanoic-phloroglucinol (90 mg, 0.14 mmol) was performed using the general procedure and afforded 3-hydroxy-5-isopropoxyphenyl docosanoate (56 mg, 82%) as white solid after purification on silica gel chromatography (hexane/AcOEt 9/1).

R_f_ (hexane/AcOEt 9.5/0.5) 0.3; ^1^H NMR (500 MHz, CDCl_3_) δ 6.22 (t, *J* = 2.2 Hz, 1H), 6.17 (t, *J* = 2.1 Hz, 1H), 6.14 (t, *J* = 2.1 Hz, 1H), 6.07 (br, 1H), 4.44 (hept, *J* = 6.1 Hz, 1H), 2.52 (t, *J* = 7.6 Hz, 2H), 1.75-1.69 (m, 2H), 1.42-1.34 (m, 4H), 1.30 (d, *J* = 6.1 Hz, 6H), 1.28 – 1.22 (m, 30H), 0.88 (t, *J* = 7.0 Hz, 3H); ^13^C NMR (126 MHz, CDCl_3_) δ 173.1, 159.8, 157.7, 152.4, 102.0, 101.9, 101.26, 70.6, 34.8, 32.3, 30.0 (9C), 30.0 (2C), 29.7, 29.8, 29.7, 29.6, 29.4, 25.2, 23.0, 22.3 (2C), 14.5.

**Synthesis of EPA-Phloroglucinol conjugate (P-EPA)**

**(5,8,11,14,17 Z)-3,5-dihydroxyphenyl icosa-5,8,11,14,17-pentaenoate**

Coupling of the di-TIPS-phloroglucinol [[13](#_ENREF_13)] (652 mg, 1.48 mmol) and EPA (543 mg, 1.79 mmol) was performed according to the general procedure and afforded the protected phloroglucinol-EPA (753 mg, 70%) as an uncoloured oil after purification on silica gel chromatography (hexane/AcOEt 99.7/0.3).

^1^H NMR (500 MHz, CDCl_3_) δ = 6.28 (t, *J*=2.1, 1H), 6.24 (d, *J*=2.1, 2H), 5.47 – 5.27 (m, 10H), 2.90 – 2.76 (m, 8H), 2.52 (t, *J*=7.6, 2H), 2.21 – 2.14 (m, 2H), 2.08 (q, *J*=7.5, 2H), 1.81 (q, *J*=7.5, 2H), 1.28 – 1.17 (m, 6H), 1.09 (d, *J*=7.3, 32H), 0.97 (t, *J*=7.5, 3H); ^13^C NMR (126 MHz, CDCl_3_) δ = 173.0, 158.5(2C), 153.2, 133.4, 130.4, 130.2, 129.9, 129.6, 129.6, 129.5, 129.5, 129.3, 128.4, 110.6, 108.2 (2C), 35.2, 27.9, 27.0 (3C), 26.9, 26.1, 21.9, 19.2 (12C), 15.7, 14.0 (6C).

Deprotection of this protected EPA-phloroglucinol (830 mg, 1.15 mmol) was performed using the general procedure and afforded the (5,8,11,14,17 Z)-3,5-dihydroxyphenyl icosa-5,8,11,14,17-pentaenoate (455 mg, 96%) as an uncoloured oil after purification on silica gel chromatography (hexane/AcOEt 9/1 to 7/3).

R_f_ (hexane/AcOEt 7/3) 0.3; ^1^H NMR (500 MHz, CDCl_3_) δ 6.09 (s, 3H), 5.46 – 5.30 (m, 10H), 2.85 – 2.80 (m, 8H), 2.57 (t, *J* = 7.5 Hz, 2H), 2.20 (q, *J* = 7.0 Hz, 2H), 2.10 – 2.05 (m, 2H), 1.83 (q, *J* = 7.5 Hz, 2H), 0.97 (t, *J* = 7.5 Hz, 3H); ^13^C NMR (126 MHz, CDCl_3_) δ 173.8, 157.6(2C), 152.0, 132.3, 129.5, 128.8, 128.8, 128.5, 128.5, 128.3, 128.3, 128.1, 127.2, 102.1(2C), 101.4, 34.0, 26.7, 25.9(3C), 25.8, 25.7, 24.8, 20.8, 14.5.

**Synthesis of LA-Phloroglucinol conjugate (P-LA)**

**Preparation of (9,12Z)-3,5-dihydroxy octadeca-9,12-dienoate**

Coupling of the mono-TIPS-mono-isopropyl-phloroglucinol [[13](#_ENREF_13)] (120 mg, 0.27 mmol) and LA (77 mg, 0.27 mmol) was performed according to the general procedure and afforded the protected mono-isopropyl-phloroglucinol-LA (120 mg, 62 %) as an uncolored oil after purification on silica gel chromatography (hexane/AcOEt 99/1).

^1^H NMR (500 MHz, CDCl_3_) δ = 6.28 (t, *J*=1.9, 1H), 6.24 (d, *J*=1.9, 2H), 5.44 – 5.27 (m, 4H), 2.78 (t, *J*=6.6, 2H), 2.50 (t, *J*=7.5, 2H), 2.12 – 1.99 (m, 4H), 1.79 – 1.66 (m, 2H), 1.43 – 1.17 (m, 20H), 1.09 (d, *J*=7.4, 32H), 0.89 (t, *J*=6.8, 3H); ^13^C NMR (126 MHz, CDCl_3_) δ = 171.9, 157.1 (2C), 151.8, 130.2, 130.0, 128.1, 127.9, 109.3, 106.9, 34.4, 31.5, 29.6 (2C), 29.4 (2C), 29.2, 29.2, 29.1, 27.2, 25.6, 24.9, 22.6, 17.9 (12C), 14.1, 12.6 (6C).

Deprotection of the protected LA-phloroglucinol 36 (59 mg, 0.08 mmol) was performed using the general procedure and afforded coumpound P-LA (30 mg, 92%) as an uncolored oil after purification on silica gel chromatography (hexane/AcOEt 8/2).

Rf (hexane/AcOEt 7/3) 0.3; ^1^H NMR (500 MHz, CDCl_3_); δ 6.06 (s, 3H), 5.45 – 5.27 (m, 4H), 2.78 (t, *J* = 6.8 Hz, 2H), 2.57 (t, *J* = 7.5 Hz, 2H), 2.10 – 2.00 (m, 4H), 1.75 (qt, *J* = 7.5 Hz, 2H), 1.46 – 1.22 (m, 14H), 0.89 (t, *J* = 6.8 Hz, 3H). ^13^C NMR (126 MHz, CDCl_3_) δ 174.6, 157.4 (2C), 151.7, 130.3, 130.0, 128.1, 127.9, 102.0, 101.5, 34.4, 31.5, 29.6 (2C), 29.4 (2C), 29.2, 29.1, 29.1, 27.2, 25.7, 24.9, 22.6, 14.1.


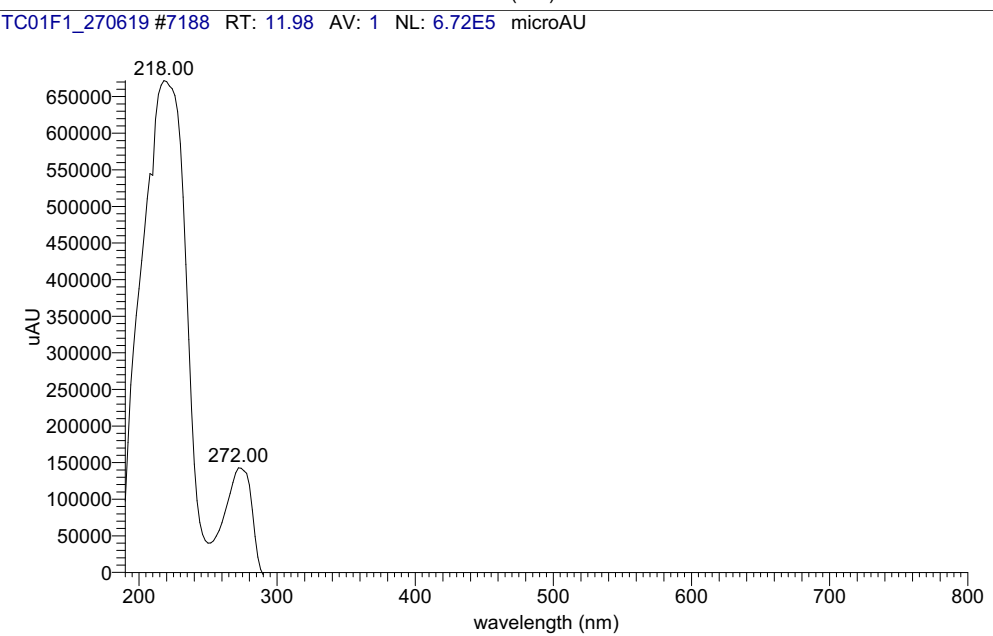


UV spectrum of IP-DHA: λ_max_ 218 nm and 272 nm.

Performed on UPLC-UV Accela from Thermo scientific, UV detector PDA (200-800nm).





**Figure S1.** Real time monitoring of the protection of ARPE-19 by IP-DHA.

The xCELLigence system allows a continuous monitoring of cell adhesion. Real-time impedance traces caused by adherent cells throughout the setting up and process of a*t*RAL cytotoxicity and IP-DHA protection are shown. The data are presented as a cell index (CI, see Material and Methods) normalized from the time of treatment. During the first 16 hours, the cells deposited and proliferated to reach subconfluency, causing a gradual steady increase in CI. When cells reach subconfluency the CI value plateaus. The addition of 25 µM a*t*RAL results in a decrease in the CI value to 11 ± 2 %. Co-incubation of a*t*RAL with 40 µM IP-DHA prevented the CI value from decreasing beyond 56 ± 11 % 2 hours after treatment and was maintained until 14 hours after medium was changed. IP-DHA alone has no effect on cell growth compared to the no-treatment CTL. The bars indicate SD of the means (n = 3).

**
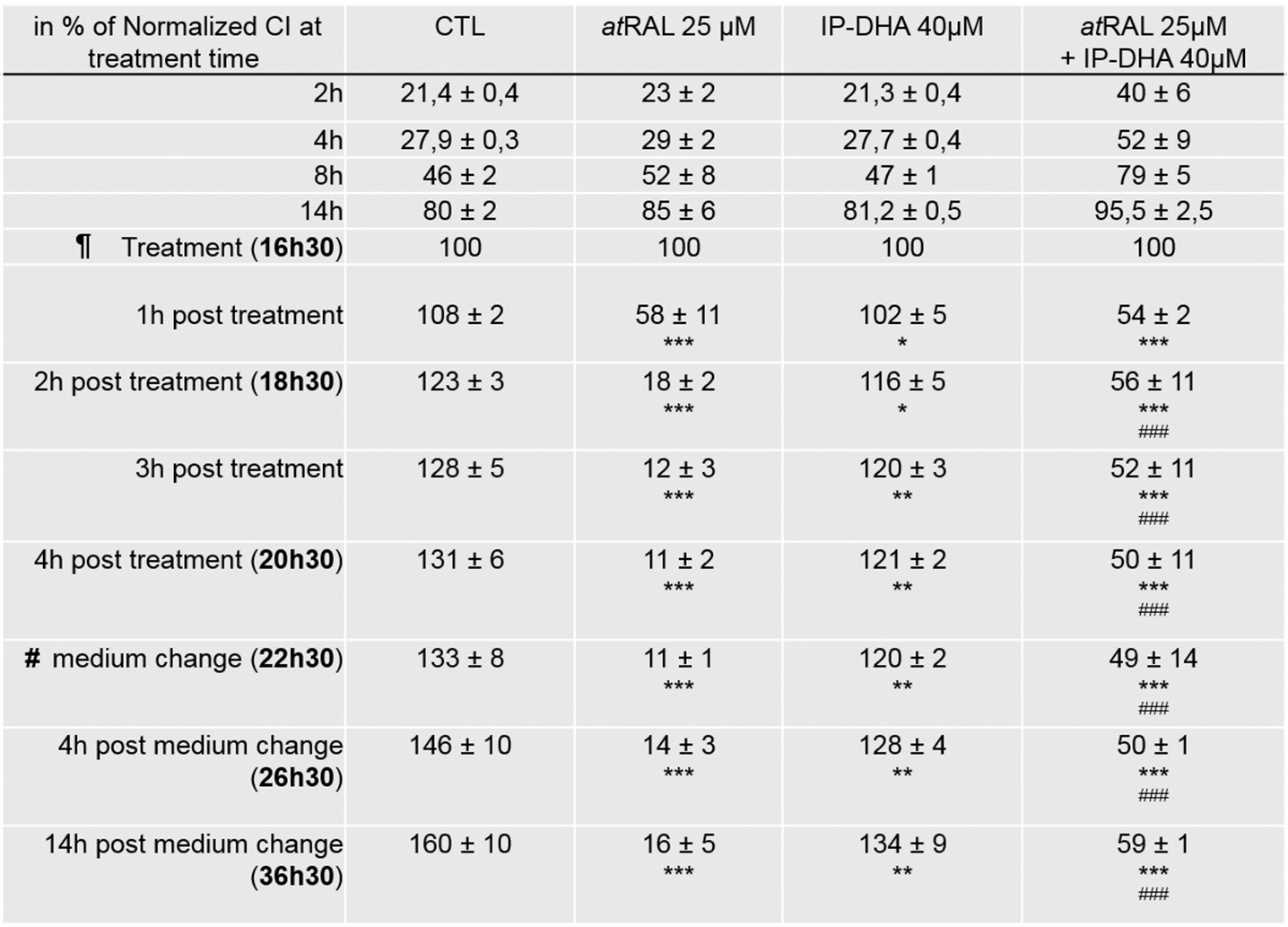
**

**Table S1.** Real time monitoring of the protection of ARPE-19 by IP-DHA.

ARPE-19 cells were plated on 16-well E-plates in growth medium until they reached subconfluency. The medium was then removed and replaced by serum-free medium alone (CTL) or containing 25 µM a*t*RAL or 40 µM IP-DHA or 25 µM a*t*RAL in the presence of 40 µM IP-DHA.

¶ Growth curves were normalized to the time point of treatment (16h30).

# Medium change was performed 6 hours post-treatment with serum-free medium (22h30).

P-value compare to CTL, * p<0.05, ** p<0.01, *** p<0.001; p-value compare to a*t*RAL, ^###^p<0.001.

**

**

**Table S2. Optimal characteristics of the chemicals showing the highest efficacy against atRAL toxicity.**
